# Effect of dietary supplementation with insect fats on growth performance, digestive efficiency and health of rabbits

**DOI:** 10.1186/s40104-018-0309-2

**Published:** 2019-01-17

**Authors:** Laura Gasco, Sihem Dabbou, Angela Trocino, Gerolamo Xiccato, Maria Teresa Capucchio, Ilaria Biasato, Daniela Dezzutto, Marco Birolo, Marco Meneguz, Achille Schiavone, Francesco Gai

**Affiliations:** 1 Department of Agricultural, Forest and Food Science, University of Turin, Largo Paolo Braccini 2, 10095 Grugliasco, Turin, Italy; 2 Department of Veterinary Science, University of Turin, Largo Paolo Braccini 2, 10095 Grugliasco, Turin, Italy; 3 Department of Comparative Biomedicine and Food Science, University of Padova, Viale dell’Università 16, 35020 Legnaro, Padova, Italy; 4 Department of Agronomy Food Natural Resources Animal and Environment, University of Padova, Viale dell’Università 16, 35020 Legnaro, Padova, Italy; 5 Veterinary Medical Research Institute for Piemonte, Liguria and Valle d’Aosta, Via Bologna 148, 10154 Turin, Italy; 6 Institute of Science of Food Production, National Research Council, Largo Paolo Braccini 2, 10095 Grugliasco, Turin, Italy; 7 Institute of Interdisciplinary Research on Sustainability, University of Turin, Via Accademia Albertina 13, 10100 Turin, Italy

**Keywords:** Blood, Digestibility, Growing rabbit, Gut health, Insect fats, Performances

## Abstract

**Background:**

The present work aimed at evaluating the effect of the dietary replacement of soybean oil (S) by two types of insect fats extracted from black soldier fly larvae (H, *Hermetia illucens* L.) and yellow mealworm larvae (T, *Tenebrio molitor* L.) on growth performance, nutrient digestibility, blood parameters, intestinal morphology and health of growing rabbits.

**Methods:**

At weaning, 200 crossbred rabbits (36 days old) were allotted to five dietary treatments (40 rabbits/group): a control diet (C) containing 1.5% of soybean oil and four experimental diets where soybean oil was partially (50%) or totally (100%) substituted by H (H50 and H100) or T (T50 and T100) fats. Total tract digestibility was evaluated on 12 rabbits per treatment. The growth trial lasted 41 d and, at slaughtering (78 days old), blood samples were collected from 15 rabbits per treatment, morphometric analyses were performed on duodenum, jejunum and ileum mucosa, and samples of liver, spleen and kidney were submitted to histological evaluation.

**Results:**

No difference was observed between the control and the experimental groups fed insect fats in terms of performance, morbidity, mortality and blood variables. The addition of H and T fats did not influence apparent digestibility coefficients of dry matter, protein, ether extract, fibre fractions and gross energy. Gut morphometric indices and organ histopathology were not affected by dietary inclusion of H and T fats.

**Conclusions:**

H and T fats are suitable sources of lipid in rabbit diets to replace soybean oil without any detrimental effect on growth performance, apparent digestibility, gut mucosa traits and health.

**Electronic supplementary material:**

The online version of this article (10.1186/s40104-018-0309-2) contains supplementary material, which is available to authorized users.

## Background

Several researches focused on the influence of the dietary supplementation of different fat sources such as animal oil or plant oilseeds [1–3] on performances, digestibility, health status, meat and fatty acid (FA) composition of rabbit meat.

The 60%–70% increase of animal products consumption expected by 2050 has pushed research to investigate new raw materials for animal feeds [4]. In this context, insects are very interesting as innovative feed for fish and terrestrial animals due to their valuable chemical composition and their claimed sustainability [5, 6]. So far, research has been mainly addressed to the protein content of insect larvae meals in fish, poultry and pigs feeds to replace conventional protein sources (mainly fishmeal and soybean meals) [7–9]. The insect species most investigated were black soldier fly (H, *Hermetia illucens* L.) and yellow mealworm (T, *Tenebrio molitor* L.) [9, 10].

Nevertheless, insects have different lipid content (ether extract from 5% to 40% of dry matter, DM) and FA profile related to the species and the rearing substrate [6, 11, 12]. When processing larvae and in order to increase the protein content and the storability of the insect processed meals, some authors underlined the importance of defatting larvae meal [13, 14]. Unlike protein meals, which use in farm animal feeding is restricted (except for fish) in Europe (Reg. EC 999/2001), there are no prohibitions on the use of insect fats as a raw material for animal compound feeds [15–17].

Concerning the insect species mentioned above, *Hermetia illucens* is a worldwide distributed Diptera which is reared for its ability to convert low value substrates into valuable products [12, 18], and whose fat is characterised by high levels of saturated fatty acids (SFA), mainly lauric (C12:0) acid [19]. *Tenebrio molitor* is a Coleoptera which larvae, considered as pests of stored grains, contain from 15% to 45% DM of fat, mostly palmitic (C14:0), oleic (C18:1 *n*-9) and linoleic (C18:2 *n*-6) acids [6, 19].

So far, the use of insect lipids in animal feeding has received little attention. Researchers investigated the effects of the dietary inclusion of H fat in partial or total replacement of soybean oil (S) without negative effects on performance and carcass traits of broiler chickens [17, 20]. Li et al. [15] evaluated the effect of H fat in substitution of up 100% S in diets for juvenile carps and did not find negative effects on growth, feed efficiency or chemical composition of fish fillets. Recently, Kierończyk et al. [16] completely substituted soybean oil with *Tenebrio molitor* or *Zophobas morio* fat (5% of dietary inclusion) in broiler diets without adverse consequences on growth performance and nutrient digestibility.

As far as rabbits are concerned, to the best of our knowledge only two old researches have been carried out using insect meal (silkworm) replacing soybean meal [21, 22] and only two research has been performed using insect fat [23, 24] in diets for growing rabbits. The aim of this research was therefore to evaluate the effect of H and T fats used as partial or total substitute of soybean oil on growth performances, digestibility, blood metabolites, intestinal morphology and histological traits of growing rabbits.

## Methods

The Ethical Committee of the University of Turin (Italy) approved the experimental protocol (Ref. 386638, 4/12/2017). The trial was carried out at the experimental rabbit facility of the Department of Agricultural, Forest, and Food Sciences (DISAFA; University of Turin, Turin, Italy).

### Diets

An experimental control diet (C) containing 1.5% soybean oil and formulated to satisfy growing rabbits requirements [25] was tested against four experimental diets where S was partially (50%) or totally (100%) substituted by H (H50 and H100) or T (T50 and T100) fats (Table 1). All diets were pelleted at the feed mill of the experimental facility at the beginning of the trial and stored in darkness and fresh to protect against lipid oxidation and degradative processes.

**Table 1:** Ingredients (g/kg as fed) and chemical composition (% DM) of experimental diets

Ingredients	Experimental diets				
	C	H50	H100	T50	T100
Dehydrated alfalfa meal (17 g CP/100 g)	320	320	320	320	320
Alfalfa hay	75	75	75	75	75
Wheat bran	235	235	235	235	235
Barley meal	100	100	100	100	100
Dried sugar beet pulp	160	160	160	160	160
Soybean meal (44 g CP/100 g)	70	70	70	70	70
Soybean oil	15	7.5	–	7.5	–
*Hermetia illucens* fat^a^	–	7.5	15	–	–
*Tenebrio molitor* fat^b^	–	–	–	7.5	15
Cane molasses	12	12	12	12	12
Dicalcium phosphate	3	3	3	3	3
Sodium chloride	4	4	4	4	4
*L*–methionine (98 g methionine/100 g)	1	1	1	1	1
Vitamin–mineral premix^c^	5	5	5	5	5
Chemical composition					
Dry matter, %	89.4	90.0	89.2	89.5	89.6
Ash, % DM	8.58	7.67	7.77	8.18	7.75
Crude protein, % DM	17.0	16.4	16.8	16.8	16.3
Ether extract, % DM	4.22	4.07	3.92	4.13	3.87
Neutral detergent fibre (aNDF), % DM	40.2	42.5	41.7	39.8	40.5
Acid detergent fibre (ADF), % DM	21.7	23.8	23.0	21.4	22.8
Acid detergent lignin (ADL), % DM	4.81	5.09	5.09	4.87	5.02
Gross Energy, MJ/kg DM	18.50	18.63	18.50	18.75	18.62

*C*, control diet; *H50* and *H100*, diets with *Hermetia illucens* fat; *T50* and *T100*, diets with *Tenebrio molitor* fat; *DM*, dry matter; *CP*, crude protein

^a^*Hermetia illucens* fat was provided by Hermetia Deutschland GmbH & Co. KG (Baruth / Mark, Germany)

^b^*Tenebrio molitor* fat was provided by Ynsect (Evry, France)

^c^Premix provided per kg of complete diet: vitamin A 16,000 IU; vitamin D_3_ 1,600 IU; vitamin E acetate 30 mg; vitamin B_1_ 0.8 mg; vitamin B_6_ 1.65 mg; niacin 40 mg; folic acid 1 mg; Mn 30 mg; Fe 116 mg; Cu 12.5 mg; Zn 60 mg; Co 0.45 g; Ca 1.3 mg; Se 0.3 mg

### Animals and experimental conditions

After weaning at 36 days of age, 200 commercial crossbred rabbits (Hycole, France) purchased from a commercial farm (Verzuolo, Cuneo, Italy), 1051 ± 138 g live weight (LW), were randomly allocated in individual wire-net cages (41 cm × 30 cm × 28 cm height). Forty rabbits, homogeneous by LW and variability, were assigned to each dietary treatment. The rabbitry had automatic control of environmental temperature (22 ± 2 °C) and photoperiod (16L:8D). The rabbits were fed ad libitum and had free access to clean drinking water during the whole trial that lasted 41 d, until 77 days of age.

At 78 days of age, rabbits were slaughtered in an experimental slaughterhouse. Data of dressing percentage, carcass traits and meat quality, and chemical composition were determined but not reported in this paper.

### Growth performance and health status

During the trial, mortality and morbidity were controlled daily by the same observer according to Gidenne et al. [26]. LW and feed intake were recorded per every rabbit on a weekly basis. At the end of the trial, the averages of LW, daily feed intake (ADFI), daily weight gain (ADG) and feed conversion ratio (FCR) were calculated.

### Digestibility trial

The apparent digestibility coefficient (ADC) and the nutritive value of experimental diets were measured during an in vivo digestibility assay performed according to the European standardised method [27]. The trial involved 60 rabbits (12 rabbits per dietary treatment) among those monitored for the fattening trial and started at 50 days of age. Faeces were daily collected per each rabbit and four consecutive days (09:00 h) and stored at − 20 °C until analyses.

### Chemical analyses and diet fatty acid profile

Diets and faeces were analysed to determine the contents of DM (method 934.01), ash (method 967.05), crude protein (CP - method 2001.11), and starch (amyloglucosidase α amylase method, 996.11) using AOAC methods [28] following harmonised procedures [29]. Ether extract (EE) was analysed after acid hydrolysis [30]. The sequential procedure and the filter bag system (Ankom Technology, Macedon, NY, USA) were used in the analyses of fibre fractions: neutral detergent fibre (aNDF) was analysed according to Mertens [31], assayed with a heat stable alfa-amylase and expressed inclusive of residual ash, without sodium sulphite; acid detergent fibre (ADF), expressed inclusive of residual ash, was analysed according to AOAC [28] (method 973.187); lignin was analysed with sulphuric acid according to Van Soest et al. [32]. Gross energy (GE) contents of diets and faeces were measured by adiabatic bomb calorimeter (IKA C200, Staufen, Germany).

The FA composition of insect lipids and feeds was determined according to Trocino et al. [33]. The fat was extracted by accelerated solvent extraction (Application Note 334; ASE®, Dionex, Sunnyvale, CA, USA) using two extraction cycles. The extracted lipids were initially trans-methylated as fatty acid methyl esters (FAMEs). An internal standard (13: 1 methyl ester) was added to the extracts before methylation. After centrifugation, the surnatant was submitted to two-dimensional gas chromatography (GC × GC) by using an Agilent 7890 A Gas Chromatograph (Agilent Technologies, Santa Clara, CA, USA). Supelco SP 2560 (Sigma-Aldrich, St. Louis, MO, USA) was used as the first capillary column (75 m × 0.18 mm internal diameter, 0.14 μm film thickness), with hydrogen as carrier. J&W HP 5 ms (Agilent Technologies) was used as the second capillary column (3.8 m × 0.5 mm internal diameter, 0.25 μm film thickness), with hydrogen as carrier. The FA were identified by comparing the retention time of standard FAMEs mixture (Supelco 37 – component FAME Mix, 47,885 – U). Individual FAMEs were expressed as the percentage of the total area of eluted FAMEs.

### Serum biochemistry parameters

At slaughtering, blood samples were collected from 15 rabbits per treatment at the moment of jugular exsanguination and put into 2.5 mL serum-separating tubes. The tubes without anticoagulant were left to clot in a standing position at room temperature for approximately two hours to obtain serum. The serum was separated by means of centrifugation at 700×*g* for 15 min and frozen at − 80 °C until analysis. The total proteins (TP) were quantified by the “biuret method” (Bio Group Medical System kit; Bio Group Medical System, Talamello, Italy). Alanino-aminotransferase (ALT), aspartate-aminotransferase (AST), alkaline phosphatase (ALP), gamma glutamyl transferase (GGT), triglycerides, cholesterol, calcium, phosphorus, iron, uric acid, urea, lactate dehydrogenase (LDH) and creatinine serum concentrations were analysed with enzymatic methods using the Screen Master Touch automated instrument (Hospitex 130 Diagnostics S.r.l., Sesto Fiorentino, Italy).

### Histomorphological variables

The same 15 rabbits per treatment used for serum biochemistry determinations were used for anatomo-pathological investigations. Segments (approximately 5 cm in length) of duodenum (after the pylorus), jejunum (middle portion) and ileum (before ileo-caecal junction) were excised and flushed with 0.9% saline to remove all the intestinal content. Samples of liver, spleen and kidney were also collected. The collected samples were fixed in 10% buffered formalin solution and submitted to morphometric analysis (intestine tracts) and histopathological examination (other organs). The tissues were routinely embedded in paraffin wax blocks, sectioned at 5 μm thickness and mounted on glass slides. Intestinal sections were submitted to haematoxylin-eosin (HE) staining, with a total of five serial sections prepared for each intestinal segment. The same slide among the serial sections was examined by light microscopy and captured with a Nikon DS-Fi1 digital camera coupled to a Zeiss Axiophot microscope using a 2.5× objective lens. NIS-Elements F software was used for image capturing. Morphometric analysis was performed by Image®-Pro Plus software. The evaluated morphometric indices were: villus height (Vh, from the villous tip to the crypt bottom), crypt depth (Cd, from the crypt bottom to the submucosa) and the villus height to crypt depth ratio (Vh/Cd; Additional file 1) [34, 35]. Morphometric measurements were performed on 10 well-oriented and intact villi and 10 crypts chosen from each gut segment.

Organ sections were submitted to HE staining and examined by light microscopy. The observed histopathological findings were evaluated using a semi-quantitative scoring system as follows: absent (score = 0), mild (score = 1), moderate (score = 2) and severe (score = 3). The following histopathological alterations were evaluated: inflammatory infiltrates and degenerative changes in liver and kidney and white pulp hyperplasia and depletion in spleen. All slides were assessed blinded by three observers and the discordant cases were reviewed at a multi-head microscope until a consensus was reached.

### Statistical analyses

The statistical analyses were performed using the IBM SPSS software package (IBM Corp. Released 2012. IBM SPSS Statistics for Windows, Version 21.0. IBM Corp, Armonk, NY). Shapiro-Wilk’s test established normality or non-normality of distribution. One-way ANOVA was used to evaluate the effect of experimental diets on growth performance, apparent digestibility coefficients, serum biochemical parameters and intestinal morphometric indices. The assumption of equal variances was assessed by Levene’s homogeneity of variance test. Mortality rate was analysed by Chi-square test, using the C group as the reference. Differences amongst groups were evaluated by the Duncan’s test. Histopathological scores were analysed by Kruskal-Wallis test (post-hoc test: Dunn’s Multiple Comparison test).

For all statistical analyses significance was declared at *P* ≤ 0.05 and a statistical trend was considered for 0.05 < *P* ≤ 0.10. The results are presented as the means and standard error of the means (SEM).

## Results

### Diet composition and fatty acid profile

Diets were comparable in terms of main crude protein (on average 16.6% DM) and gross energy contents (18.6 MJ/kg DM), despite some differences in aNDF and ADF contents (Table 1).

The substitution of S with insect fats modified the FA profile of the lipids in the diets (Table 2). In fact, lauric acid (C12:0) and, thus, total SFA rate were the highest in H fat and H diets; oleic acid (C18:1 *n*-9) and MUFA rates were the highest in T fat and T diets; whereas, linoleic acid (C18:2 *n*-6) and PUFA dominated the FA profile of S and C diets.

**Table 2:** Fatty acids profile (% of total FA) of dietary fats and experimental diets

Fatty acids	Dietary fats			Experimental diets				
	S	H	T	C	H50	H100	T50	T100
C12:0	0.02	48.0	0.23	0.05	9.12	20.3	0.75	0.30
C14:0	0.08	10.3	2.22	0.09	2.11	4.47	0.97	1.33
C16:0	10.4	12.7	17.6	12.1	15.7	16.1	17.3	18.4
C18:0	4.43	1.90	2.31	2.84	2.62	2.08	2.65	2.22
C16:1 *n*-7	0.09	3.20	1.66	0.12	1.31	1.99	0.70	1.04
C18:1 *n*-9	23.0	9.11	37.8	20.1	17.3	12.7	24.6	27.3
C18:2 *n*-6	51.5	9.00	33.2	52.1	40.9	31.0	42. 9	38.9
C18:3 *n*-3	7.03	1.01	1.80	7.43	6.79	6.28	5.29	5.51
SFA^1^	15.8	74.8	23.1	16.5	31.5	45.4	23.4	24.0
UFA^1^	84.2	25.2	76.9	83.5	68.5	54.6	76.6	76.0
MUFA^1^	25.4	14.1	41.1	23.6	20.5	16.9	27.9	30.9
PUFA^1^	58.8	11.1	35.8	59.9	48.0	37.7	48.7	45.1

^1^Included minor FAs

*S* soybean oil, *H Hermetia illucens* fat, *T Tenebrio molitor* fat, *C* control diet; *H50* and *H100* diets with *Hermetia illucens* fat; *T50* and *T100* diets with *Tenebrio molitor* fat, *MUFA* monounsaturated fatty acid, *PUFA* polyunsaturated fatty acid, *SFA* saturated fatty acid, *UFA* unsaturated fatty acid

### Growth performance

Table 3 reports the effects of the experimental diets on growth performance. After 41 d on trial, no differences were observed among the control and the experimental groups in terms of performance, morbidity and mortality. The final LW ranged from 2811 g (diet T50) to 2917 g (diet T100). The ADFI, ADG and FCR were 152 g/d, 44.5 g/d and 3.44, respectively, on average for the five groups.

**Table 3:** Growth performance of rabbits fed the experimental diets (*n* = 40 rabbits/group)

Items	Experimental diets					SEM	*P*-value
	C	H50	H100	T50	T100		
Number of rabbits	40	40	40	39	38		
Initial live weight, g	1039	1059	1068	1030	1057	9.7	0.74
Final live weight, g	2907	2859	2880	2811	2917	21.3	0.54
ADG, g/d	45.6	43.9	44.2	43.5	45.1	0.43	0.53
ADFI, g/d	154	149	153	149	154	1.2	0.55
FCR	3.40	3.44	3.52	3.45	3.41	0.02	0.38
Mortality, %	0	0	0	2.5	5.0	–	0.99
Morbidity, %	27.5	22.5	27.5	25.0	22.5	–	0.86

*C* control diet, *H50* and *H100* diets with *Hermetia illucens* fat, *T50* and *T100* diets with *Tenebrio molitor* fat, *SEM* standard error of the means, *ADG*, daily weight gain, *ADFI* daily feed intake, *FCR* feed conversion ratio

### Digestibility trial

The ADC are reported in Table 4. The addition of H and T fats did not influence the digestibility of nutrients and energy. Only the digestibility of ADF tended to be lower in T diets compared to the control diet (22.5% on average vs. 26.4%; *P* = 0.08).

**Table 4:** Rabbit feed intake and apparent digestibility coefficients during the digestibility trial (*n* = 12 rabbits/group)

Items	Experimental diets					SEM	*P*-value
	C	H50	H100	T50	T100		
Feed intake, g/d	158	151	159	155	155	2.55	0.87
Dry matter, %	61.6	60.8	60.6	60.9	61.6	0.30	0.78
Organic matter, %	61.2	60.6	60.5	60.8	61.4	0.31	0.84
Crude protein, %	71.9	72.1	72.9	71.0	72.5	0.29	0.35
Ether extract, %	82.6	82.7	82.2	80.9	81.9	0.38	0.57
aNDF, %	33.5	32.0	32.2	32.0	30.2	0.50	0.44
Hemicelluloses, %	41.7	39.7	42.4	42.6	40.6	0.49	0.27
ADF, %	26.4	26.0	24.0	22.9	22.2	0.54	0.08
Energy, %	60.7	60.4	59.9	60.8	61.0	0.30	0.83
Nutritive value							
DP, g/kg	109.3	105.5	109.7	106.6	105.5	–	–
DE, MJ/kg	10.0	10.1	9.9	10.2	10.2	–	–
DP/DE ratio, g/MJ	10.9	10.5	11.1	10.5	10.4	–	–

*C* control diet, *H50* and *H100* diets with *Hermetia illucens* fat, *T50* and *T100* diets with *Tenebrio molitor* fat, *SEM* standard error of the means, *aNDF* Neutral detergent fibre, *ADF* Acid detergent fibre, *DP* digestible protein, *DE* digestible energy; *DP/DE ratio,* digestible protein to digestible energy ratio

### Serum biochemistry parameters

The different dietary H or T fat inclusion levels did not influence (*P* > 0.10) the serum biochemical parameters of the rabbits (Table 5). Only serum TP, cholesterol, LDH and phosphorus tended to differ among diets (*P* ≤ 0.10).

**Table 5:** Effect of lipid sources on serum biochemical traits of rabbits fed experimental diets (*n* = 15 rabbits/group)

Items	Experimental diets					SEM	*P*-value
	C	H50	H100	T50	T100		
TP, g/dL	5.4	5.1	5.1	5.1	5.1	0.04	0.08
AST, IU/L	8.9	12.2	9.8	8.8	10.2	0.51	0.20
ALT, IU/L	42.5	43.1	45.2	47.2	40.3	1.13	0.38
ALP, IU/L	34.7	43.7	32.2	45.6	39.7	2.83	0.53
GGT, IU/L	49.0	38.7	57.9	50.5	56.6	2.75	0.19
Uric acid, mg/dL	1.5	1.4	1.5	1.5	1.5	0.03	0.99
Urea, mg/dL	18.0	17.2	14.7	15.9	17.7	0.87	0.76
Creatinine, mg/dL	0.6	0.7	0.8	0.8	0.8	0.04	0.53
Triglycerides, mg/dL	123.0	122.8	120.4	126.1	103.1	6.79	0.84
Cholesterol, mg/dL	47.4	52.6	45.7	38.3	51.4	1.82	0.10
LDH, IU/L	143.5	146.9	120.7	199.9	117.4	10.4	0.09
Iron, μg/dL	293.0	60.8	242.1	289.3	312.2	13.1	0.47
Calcium, mg/dL	9.5	11.2	10.0	9.9	10.2	0.39	0.73
Phosphorous, mg/dL	6.8	7.6	9.3	7.7	6.6	0.34	0.08

*C* control diet, *H50* and *H100* diets with *Hermetia illucens* fat, *T50* and *T100* diets with *Tenebrio molitor* fat, *SEM* standard error of the means, *TP* total proteins, *AST* aspartate-aminotransferase, *ALT* alanine-aminotransferase, *ALP* alkaline phosphatase, *GGT* Gamma-glutamyl transferase, *LDH* lactic acid dehydrogenase

### Histomorphological investigations

Villi height, crypt depth and their ratio were not affected by the experimental treatment (Table 6).

**Table 6:** Effects of lipid sources on the gut morphometric indices of rabbits (*n* = 15 rabbits/group)

Items	Experimental diets					SEM	*P*-value
	C	H50	H100	T50	T100		
Duodenum							
Vh, mm	1.69	1.43	1.58	1.67	1.59	0.08	0.56
Cd, mm	0.09	0.09	0.08	0.09	0.09	0.004	0.92
Vh/Cd	18.55	16.95	18.60	20.73	18.14	0.64	0.49
Jejunum							
Vh, mm	1.53	1.50	1.69	1.68	1.63	0.07	0.51
Cd, mm	0.09	0.08	0.08	0.08	0.08	0.003	0.99
Vh/Cd	18.76	18.03	22.29	20.24	20.15	0.77	0.51
Ileum							
Vh, mm	1.02	1.35	1.24	1.40	1.25	0.08	0.55
Cd, mm	0.08	0.09	0.09	0.08	0.09	0.004	0.80
Vh/Cd	13.47	15.77	14.85	17.27	15.17	0.46	0.12

*C* control diet, *H50* and *H100* diets with *Hermetia illucens* fat, *T50* and *T100* diets with *Tenebrio molitor* fat, *SEM* standard error of the means, *Vh* villi height, *Cd* crypt depth, *Vh/Cd* villus height to crypt depth ratio

Histopathological alterations were present in all the organs of all rabbits regardless from the dietary treatments (Table 7). Multifocal to diffuse (especially centrilobular) vacuolar degeneration of the hepatocytes and focal to multifocal periportal and/or interstitial lymphoplasmacytic inflammation were identified in liver (Additional file 2). The spleen showed multifocal to diffuse white pulp hyperplasia or depletion. Finally, focal to multifocal interstitial lymphoplasmacytic inflammation was observed in kidney, with no degenerative changes being identified. Dietary inclusion of H or T fats did not affect the severity of the histopathological alterations (*P* > 0.05).

**Table 7:** Effects of lipid sources on the histopathological scores of rabbits (*n* = 15 rabbits/group)

Items	Experimental diets					SEM	*P*-value
	C	H50	H100	T50	T100		
Liver							
Inflammation score	1.21	1.21	0.88	0.67	0.79	0.11	0.42
Degeneration score	1.46	1.79	1.33	1.17	1.64	0.12	0.35
Kidney							
Inflammation score	0.64	0.79	0.67	0.50	0.57	0.11	0.91
Degeneration score	Absence of degenerative changes						
Spleen							
White pulp hyperplasia	0.14	0.14	0.00	0.00	0.14	0.05	0.79
White pulp depletion	0.29	0.14	0.00	0.17	0.29	0.07	0.71

*C* control diet, *H50* and *H100* diets with *Hermetia illucens* fat, *T50* and *T100* diets with *Tenebrio molitor* fat, *SEM* standard error of the means

## Discussion

Only few papers investigated the effects of insect oils in poultry feeds [16, 17, 20] and only two deals with the inclusion of insect fat in growing rabbit diets [23, 24]. The results obtained in this study are consistent with the hypothesis enunciated in the background and showed how the dietary insect oil inclusion did not negatively affected performances, digestibility, gut mucosa and rabbit health.

### Growth performance and nutrient digestibility

At the end of the growing period, no statistical differences among groups were reported for the growth performance traits. During the trial, the rabbits were not treated with antibiotics and the mortality was low and not influenced by dietary treatment. The similar feed intake indicated that 0.75% and 1.50% inclusion levels of both insect fats were acceptable to growing rabbits. A preference test where poultry were free to choose between diets containing H or S fats did not reveal any difference, confirming a good acceptability of both insect lipid sources [20]. Our data are fully consistent with those reported by Martins et al. [25] who observed unaffected growth performances in growing rabbits fed H fat with two inclusion level of supplementation in comparison to two inclusion levels of extruded linseed. Consistently with our results, in poultry, Schiavone et al. [17, 20] reported that partial or full replacement of S with H fat had no effect on birds growth performance or health. Similarly, the use of 5% of T or *Zophobas morio* fats in total substitution of S in a 28-day trial did not affect the growth and feed efficiency of broiler chickens [16].

As far as fish are concerned, Li et al. [15] studied the effect of increasing the replacement rate of S with H fat on juvenile Jian carp (*Cyprinus carpio* var. Jian) and reported no differences on growth performance and nutrient utilization. Recently, Dumas et al. [36] evaluated up to 10% of H fat dietary inclusion in partial or total substitution of fish oil in diets for rainbow trout and reported no significant effect on final body weight, weight gain, FCR and mortality.

Other researches using different (animal and vegetable) oils and fats in rabbit diets did not find significant differences in growth performance, indicating that rabbit can digest and metabolize different lipid sources, at least until 3% of inclusion level [3]. For instance, both Rodríguez et al. [37] and Kowalska and Bielanski [38] found similar growth performance and feed conversion efficiency when feeding rabbits with lard fat, fish or soybean oils. Also, Trebušak et al. [39] showed similar growth performance in rabbits fed diets containing palm fat (rich in SFA) or linseed oil (rich in PUFA). Peiretti et al. [40] did not find differences in growth performance of rabbits fed diets supplemented with maize or palm oil. The lack of an effect of the lipid source on growth performance was also reported by Chen and Li [41] when lard was added to rabbit diets instead of S, as well as by Casado et al. [42] feeding fattening rabbits with diets containing 3% of animal fat (lard), vegetable sources (sunflower or linseed oils) or a 1:1 mixture of sunflower oil and lard or linseed oil and lard. Conversely, Djakalia et al. [43] reported higher performance in growing rabbits fed S diet compared to those fed palm oil diet and ascribed this effect to the higher PUFA and lower SFA levels of the former. Differences among studies could depend more on the different fat levels tested [2] than the lipid source [3]. Indeed, in our trial, dietary fat level was maintained around standard values for growing rabbits [25]. Likely, at higher inclusion levels, differences in FA profile among the different added fat could have caused large differences in fat digestive utilization and thus in growth [2, 3].

To go in deep on the evaluation of dietary treatments on nutrient digestibility, in the present trial DM and energy digestibility coefficients were high and similar in all treatments indicating that both insect lipid sources are suitable for rabbit feeding. As the digestibility of EE is concerned, the mean value was consistent with literature data. Differences may depend on the type of fat source, their unsaturation degree as well as the level of structural lipids linked to cell walls [3, 44, 45]. In fact, the digestibility of more saturated fats, like tallow and lard, may be lower than that of unsaturated fat, like sunflower and soybean oils [46]. However, in the current study, the higher levels of SFA of H diets, did not determine EE digestibility lower than C or T diets. Similarly, in diets for rainbow trout, Dumas et al. [36] did not find significant differences for EE digestibility when 20% H fat was added to diets. Also, Chen and Li [41] did not observe significant difference in the EE digestibility depending on the FA profile of the lipid source (lard vs soybean oil) in diets for growing rabbits.

In the contrary, Martins et al. [24] reported that H fat with two inclusion level in growing rabbit’s diet decreased the ADC of DM, OM, EE and GE when compared to linseed fat source. The authors found that increasing the level of H fat in the diets led to a significant increase in the ADC of EE and a significant decrease in the ADC of the cellulose.

### Serum biochemistry traits

In the current trial, the values of the different traits lay within the normal range for rabbits [47–49]. This suggests that the rabbits were within physiological conditions. In our trial, only the type of fat included were different and diets had the same nutrient levels. This could justify the lack of significant differences in TP values between groups. Indeed, Iyayi and Tewe [50]. reported that serum TP synthesis is generally related to the content of available protein in the diet. Including insect lipids in rabbit diets did not influence AST, ALT and ALP enzyme activities, which are considered to be important indicators of liver functions [51].

Indeed, also in broiler chickens, Schiavone et al. [17, 20] showed that dietary partial or total replacement of S with H fat did not affect serum biochemistry parameters. The same finding was reported with Li et al. [15] in juvenile Jian carp fed diets with different substitution levels of S with H fat.

### Histomorphological investigations

Since both gut morphology and growth performance were unaffected in H and T groups of the current research, insect lipids utilization did not negatively influence gut development and functionality. Consistently with the results of the present study, Schiavone et al. [17] observed unaffected intestinal morphometric indices in broiler chickens fed with H fat replacing 50% or 100% of S.

Dietary H or T fat inclusion did not affect either the development or the severity of the histopathological alterations in the rabbits of the current research. Degenerative and inflammatory changes of liver and kidney are usually observed in toxic diseases [52] or toxicity-induced oxidative stress conditions [53]. Hepatic and renal oxidative stress development has also been reported in rabbits fed with hypercholesterolemic diets [54], thus underlying the impact of dietary lipid modifications on liver and kidney functionality. However, no hypercholesterolemia was observed in H and T rabbits of the present study. Furthermore, the majority of the histopathological alterations varied from absent to moderate in all the dietary treatments and they were also identified in the animals fed with C diet, thus allowing to reliably exclude a potential influence of insect lipids utilization in their development.

## Conclusions

The obtained results showed that H and T fats can be used as partial or total substitute of soybean oil until 1.5% of the diet in rabbit feeding without impacting growth performance, nutrient digestibility, serum biochemical traits and gut development. These findings suggest that H and T fats is a suitable ingredient for rabbit diets. However, further studies should be performed to establish the most effective source and to determine the optimal inclusion level of insect lipids for rabbit feeding.

## Additional files

Additional file 1: Morphometric evaluation of the jejunum segment of the rabbits. Morphometric measurements of the villus height (Vh) and the crypt depth (Cd). H100 group, 2.5× haematoxylin-eosin stain. (TIF 7150 kb)

Additional file 2 Histopathological findings of the rabbits. (A) HI50 group. A normal liver is observed. 10× haematoxylin-eosin stain. (B) H100 group. Liver, periportal zone. Moderate and multifocal vacuolar degeneration of the hepatocytes (arrowheads), as well as mild and multifocal lymphoplasmacytic inflammation (arrows), are identified. 10× haematoxylin-eosin stain. (TIF 5050 kb)

## Abbreviations

ADC: Apparent digestibility coefficient
ADF: Acid detergent fibre
ADFI: Daily feed intake
ADG: Daily weight gain
ADL: Acid detergent lignin
ALP: Alkaline phosphatase
ALT: Alanine amino-transferase
aNDF: Neutral detergent fibre
AST: Aspartate amino-transferase
C: Control diet
Cd: Crypt depth
CP: Crude protein
DE: Digestible energy
DM: Dry matter
DP: Digestible protein
EE: Ether extract
FA: Fatty acid
FAME: Fatty acids methyl esters
FCR: Feed conversion ratio
GE: Gross energy
GGT: Gamma-glutamyl transferase
H: *Hermetia illucens*
HE: Haematoxylin-eosin
HR_i_: Health risk index
LDH: Lactic acid dehydrogenase
LW: Live weight
MUFA: Monounsaturated fatty acid
PUFA: Polyunsaturated fatty acid
S: Soybean oil
SEM: Standard error of the means
SFA: Saturated fatty acid
T: *Tenebrio molitor*
TP: Total proteins
UFA: Unsaturated fatty acid
Vh / Cd: Villus height to crypt depth ratio
Vh: Villus height
