# Supplementary figures and images for: Effect of dietary supplementation with insect fats on growth performance, digestive efficiency and health of rabbits
Source: J Anim Sci Biotechnol. 2019 Jan 17;10:4. doi: 10.1186/s40104-018-0309-2 (PMC6337837; doi:10.1186/s40104-018-0309-2)

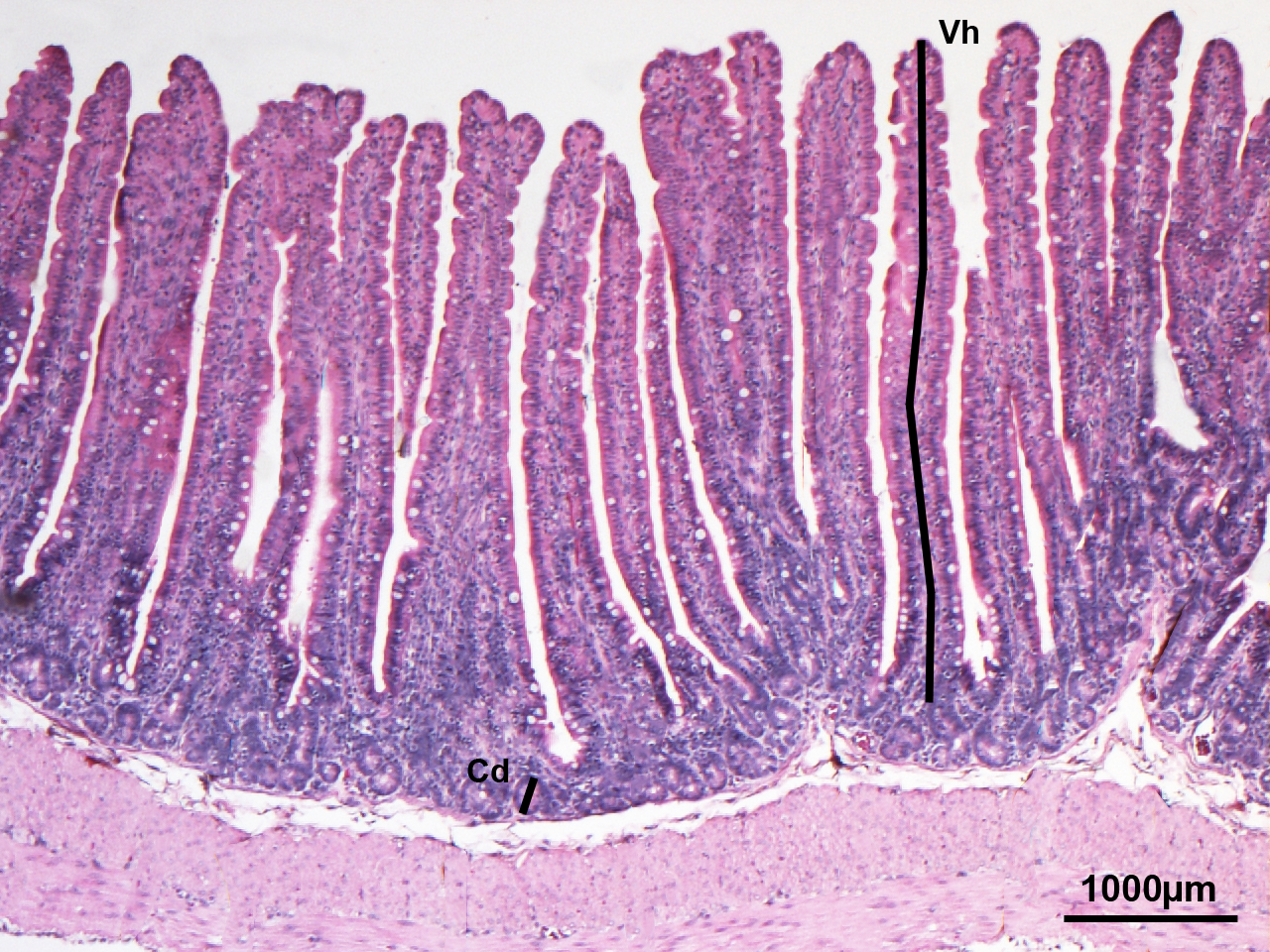

Supplement: Supplementary file 1 — Morphometric evaluation of the jejunum segment of the rabbits. Morphometric measurements of the villus height (Vh) and the crypt depth (Cd). H100 group, 2.5× haematoxylin-eosin stain. (TIF 7150 kb) [file 40104_2018_309_MOESM1_ESM.tif]

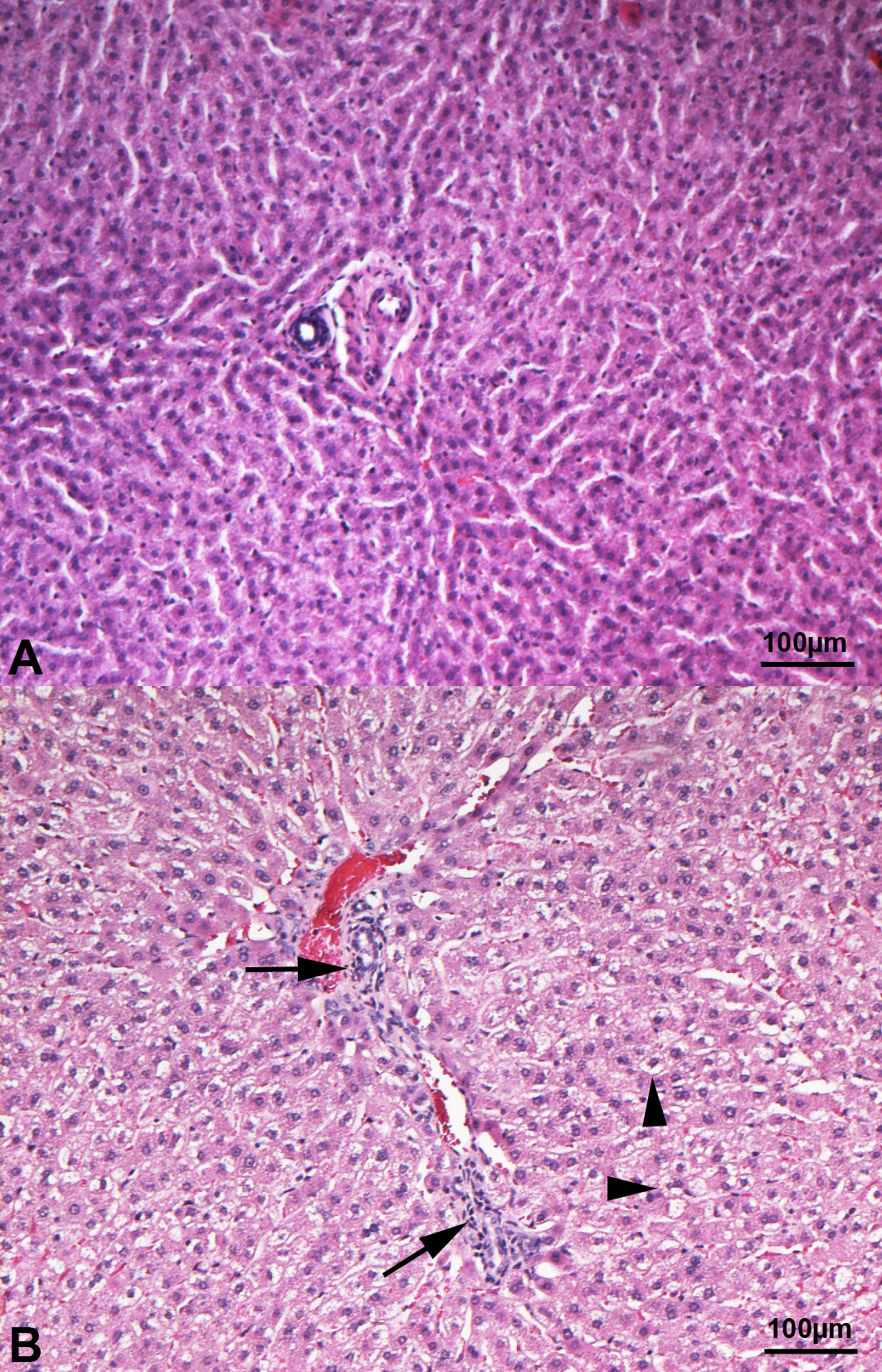

Supplement: Supplementary file 2 — Histopathological findings of the rabbits. (A) HI50 group. A normal liver is observed. 10× haematoxylin-eosin stain. (B) H100 group. Liver, periportal zone. Moderate and multifocal vacuolar degeneration of the hepatocytes (arrowheads), as well as mild and multifocal lymphoplasmacytic inflammation (arrows), are identified. 10× haematoxylin-eosin stain. (TIF 5050 kb) [file 40104_2018_309_MOESM2_ESM.tif]
